# The Time for Race-Neutral Pulmonary Function Norms is Now

**DOI:** 10.1089/heq.2023.0274

**Published:** 2024-09-30

**Authors:** Peter H. S. Sporn, Cheryl Conner, Min J. Joo, Bijal Jain, Sheryl Lowery, Natasha Nichols

**Affiliations:** ^1^ Jesse Brown Veterans Affairs Medical Center, Northwestern University Feinberg School of Medicine, Chicago, Illinois, USA.; ^2^ Jesse Brown Veterans Affairs Medical Center, University of Illinois College of Medicine, Chicago, Illinois, USA.; ^3^ Jesse Brown Veterans Affairs Medical Center, University of Illinois College of Pharmacy, Chicago, Illinois, USA.

Dear Editor:

We applaud the journal and guest editors on publication of the recent issue, *Special Collection: Race in Clinical Algorithms*. The articles in the special issue illuminate the ways in which racial biological essentialism permeates biomedicine, promotes the misuse of race in clinical decision-making, and contributes to inequities in health care and health outcomes. As members of the Clinical Algorithms Committee of the Jesse Brown for Black Lives (JB4BL) Task Force at the Jesse Brown Veterans Affairs Medical Center (VAMC) in Chicago, we write in response to the article by List et al, “Eliminating Algorithmic Racial Bias in Clinical Decision Support Algorithms: Use Cases from the Veterans Health Administration.”^1^ The authors state that the Veterans Health Administration (VHA) “uses equity- and evidence-based principles to examine, correct, and eliminate use of potentially biased clinical equations and predictive models.” However, we believe that VHA national leadership has fallen short of this stated purpose in the approach it has so far taken to address the misuse of race in pulmonary function test interpretation.

The concept that race is a biological determinant of lung function has its early origin in measurements of vital capacity in southern White people and enslaved Black people by the plantation physician Samuel Cartwright, who claimed that pulmonary “deficiency” in Black people justified their enslavement and forced labor.^2^ Studies in the modern era, including the third National Health and Nutrition Examination Survey (NHANES III),^3^ have shown lower lung function in Black as compared with White Americans, and race-specific reference equations based on NHANES III have been widely used in pulmonary function test interpretation. However, it has long been known that pulmonary function varies much more within racial and ethnic groups than it does between them. Moreover, accumulating evidence from recent studies shows that race-neutral reference equations better predict mortality and incident chronic pulmonary disease in population-based cohorts,^4,5^ and symptoms, functional capacity and emphysema in subjects with chronic obstructive pulmonary disease (COPD).^6^ It has also been shown that the prevalence of emphysema in specific ranges of percent predicted forced expiratory volume at one second (FEV1) is better matched between Black and White persons with use of race-neutral rather than race-specific reference equations.^7^ Taken together, these studies indicate that race-specific reference equations mask differential exposures that reduce lung function and misrepresent poor lung health as normal or less severe than it actually is in Black individuals.

Based on an exhaustive review of the evidence, including the aforementioned studies, in April 2023 experts representing the American Thoracic Society (ATS) Committees on Pulmonary Function Testing and on Health Equity and Diversity published an official ATS guideline that recommended replacing race and ethnicity-specific equations with race-neutral average reference equations for interpretation of pulmonary function test results.^8^ In making this recommendation, the authors cited multiple benefits of using race-neutral equations, including

a) Recognition that race and ethnicity are social constructs, not biological variables;
b) Consistency with the scientific evidence that an average reference standard best predicts mortality, incident pulmonary disease, and symptoms and lung structure in COPD; and
c) Greater likelihood that Black and other persons of color with results near thresholds would undergo further evaluation for pulmonary disease and that they could meet criteria for pulmonary rehabilitation, noninvasive ventilatory support, and early referral and listing for lung transplantation or lung volume reduction surgery.

In short, moving from race-specific to race-neutral reference equations has the potential to reduce inequities in care and improve outcomes in persons of color who have historically been harmed by under-diagnosis and under-treatment of lung disease. Another benefit of abandoning race-specific reference equations is that it avoids accepting differences in pulmonary function as biologically intrinsic to race and prompts a shift in focus to determine the exposures and adverse influences on respiratory health due to systemic racism that account for such differences.

The focus of the Jesse Brown VAMC Clinical Algorithms Committee is on critical evaluation of the use of race in clinical algorithms and on removing race from such algorithms when potential harms to our veteran patients are evident and when inclusion of race is not scientifically justified. Based on this principle, and on our Committee’s evaluation of the evidence against use of race-specific pulmonary function norms (prior to publication of the official ATS guideline), the Pulmonary Function Lab at Jesse Brown VAMC transitioned to using race-neutral pulmonary function reference equations on August 1, 2022. Further, representatives of our Committee advocated to the clinical leadership of our regional VA hospital network (Veterans Integrated Service Network [VISN] 12) that all facilities in the network switch from race-specific to race-neutral reference equations. We presented the evidence in support of the move to race-neutral equations to the VISN 12 Healthcare Delivery Council in November 2022 and solicited input from pulmonary function lab directors within the network in January 2023. As a result, our proposal was unanimously approved in March 2023. Since this time, six of eight VISN 12 health care facilities have implemented race-neutral pulmonary function reference equations.

While we have put in place race-neutral pulmonary function reference equations in VISN 12, we are troubled that the VA National Program Office for Critical Care, Pulmonary, and Lung Cancer Screening has deferred adopting this approach for VA nationwide. Instead, List et al. indicate that a “multi-step approach” involving data collection within the VA and focus groups to engage veteran opinions about changing to race-neutral equations will be undertaken. This approach ignores the fact that race is not a biological determinant of pulmonary function and defies the scientific evidence that race-specific equations fail in predicting important clinical outcomes and misrepresent poor lung health as normal in Black people. Thus, we strongly urge the VA National Program Office to reconsider its position and implement race-neutral reference equations across the VA system nationally in accord with the ATS guideline, as we have done in VISN 12.

We recognize that the transition to race-neutral prediction equations will have consequences and present challenges beyond those discussed above. These include potential negative impacts on determining eligibility for surgical resection in Black patients with lung cancer and on eligibility for disability benefits among white veterans with lung disease. These issues call for new approaches to evaluation of surgical risk and disability assessment that incorporate measures of physiology and functional ability beyond standard pulmonary function tests. However, they are not reasons to delay abandoning an obsolete and scientifically unjustified approach to pulmonary function test interpretation that contributes to health inequities resulting in demonstrable harm to Black and other veterans of color.

## Abbreviations Used

ATS: American Thoracic Society
COPD: chronic obstructive pulmonary disease
FEV1: forced expiratory volume at one second
JB4BL: Jesse Brown for Black Lives Task Force
NHANES III: National Health and Nutrition Examination Survey
VA: Veterans Affairs
VAMC: Veterans Affairs Medical Center
VHA: Veterans Health Administration
VISN: Veterans Integrated Service Network
